# Two Regional Deployment Algorithms of Distributed GNSS Forwarding Spoofer for Multiple Receiver Sensors

**DOI:** 10.3390/s22207793

**Published:** 2022-10-14

**Authors:** Yangjun Gao, Guangyun Li

**Affiliations:** 1 College of Geospatial Information, PLA Strategic Support Force Information Engineering University, Zhengzhou 450001, China; 2 State Key Laboratory of Geo-Information Engineering, Xi’an 710054, China

**Keywords:** multiple receiver sensors, forwarding spoofing, distributed spoofing, regional deployment, GNSS

## Abstract

Global navigation satellite system (GNSS) spoofing technology is an effective way to protect sensitive facilities and control foreign objects. To realize effective distributed GNSS forwarding spoofing for multiple receiver sensors in the area, the following research work is carried out: first, the GNSS forwarding spoofing model is established, including a forwarding spoofing mathematical model and an asynchronous traction spoofing method; second, the function scope of forwarding spoofing is fully evaluated; third, two forwarding spoofer area deployment algorithms for multi-spoofer multi-target (MSMT) are proposed—the multi-target same-point spoofing algorithm (MSPSA) is suitable for the same-point spoofing of multiple receiver sensors, and the multi-target different-point spoofing algorithm (MDPSA) is suitable for the different-point spoofing of multiple receiver sensors; and four, the experimental tests of MSPSA and MDPSA for MSMT were carried out. The experimental results show that MSPSA and MDPSA can respectively give the most appropriate deployment scheme of spoofing according to the various needs of the spoofer. For example, the number of spoofing devices is 1, the total distance between each spoofer and each receiver is 40,345.1 m, and the critical spoofing rate (CSR) and spoofing success rate (SSR) are both 100%. The performance of the spoofing deployment scheme can meet the needs of the spoofer.

## 1. Introduction

At present, the major global navigation satellite systems (GNSS) are in the stage of rapid development and stable operation. Thanks to the steadily improved service performance of GNSS, the vast number of GNSS users can easily obtain high-precision, all-weather, and continuous stable positioning, navigation, and timing (PNT) services. On the other hand, a GNSS navigation signal, as a radio signal from a long-distance navigation satellite, has certain vulnerabilities, such as low signal power and partial signal structure disclosure. Based on this, a jammer can carry out complex and changeable signal interference and even spoofing to the ground, low altitude, and even aviation GNSS users [1] to achieve the tactical and even strategic intent of driving away unknown aircraft and protecting sensitive parts [2,3]. For the jammer, the harmfulness and concealment of GNSS spoofing are far better than other forms of interference [4,5], so GNSS spoofing has gradually become a navigation-jamming technology favored by jammers [6]. Many spoofing methods have emerged and can affect various navigation terminals [2,4]. For different spoofed navigation terminals and purposes of spoofing, the forwarding spoofing adds a specific delay to each GNSS signal and reasonably deploys the distributed spoofing devices to achieve the purpose of spoofing to the maximum extent. Forwarding spoofing can be used to spoof the military code receiver sensor. This advantage is difficult to achieve by generating spoofing, so its application prospect is broad.

The delay control of a retransmitted signal is one of the key technologies of forwarding spoofing. Huisuo Zhang et al. implemented the algorithm of simultaneous spoofing of position and velocity by applying different propagation delays and Doppler control quantities on each satellite to slowly pull the GNSS positioning results [7]. Shangyue Wang et al. adopted an iterative algorithm and introduced an offset rate parameter to add delay to the GNSS signal to realize the forwarding spoofing scheme of point-by-point deviation of a user’s track. A simulation shows that the user does not have location jump when being cheated, and the spoofing effect is obvious [8]. In addition, Jiaqi Zhang et al. proposed an intelligent forwarding spoofing scheme based on the low Earth orbit (LEO) satellite platform, improving the signal spatial separation algorithm, which was more suitable for the fast-moving LEO satellite platform [9]. The main contribution of the above research is that the position and velocity spoofing of the victim receiver can be realized through a flexible time-delay-control algorithm.

On the other hand, it is difficult to accurately control the carrier Doppler and code phase of the retransmitted signal for forwarding spoofing, so it is necessary to control the transmission power of the retransmitted signal to achieve effective spoofing. Through simulation analysis, Yanbin Liu et al. found that when the forwarding gain does not exceed 30 dB and the forwarding space delay exceeds 1 chip, there is almost no correlation interference between the retransmitted signal and the direct signal; when the retransmitted signal and the direct signal coexist, the retransmitted signal only needs to have 7~10 dB power gain to have a high acquisition probability [10]. Pengliang Shi et al. pointed out that when the retransmitted signal is 30 dB higher than the direct signal, the carrier tracking loop will lose lock, and the receiver will track the spoofing signal with a large probability after recapture [11]. Although the above research results are only for the case of spoofing a single victim receiver, the main contribution is, first, as the theoretical basis for the spoofing multiple receivers below, and, second, it is also the theoretical basis for the following analysis of the scope of forwarding spoofing.

The research results of distributed deployment of forwarding spoofing are summarized as follows. The angle of arrival detection algorithm is one of the effective spoofing detection methods to deal with spoofing signals broadcast from a single spoofing device [12]—for example, the antenna-array carrier phase double-difference detection method [13], inertial navigation-assisted three-element antenna-array detection method [14], and Doppler-based spoofing source-direction-finding method [15]. Jingshu Yang et al. used multiple directional antennas and delay controllers to control the delay of different satellite signals to achieve position spoofing of the target receiver, established a four-station forwarding spoofing model, and gave the mapping relationship between the real position and the spoofing position [16]. Tippenhauer et al. pointed out that it is applicable to the forwarding spoofing of military code signals, and a spoofer is limited to fewer specific spatial positions [17]. Ting He proposed to lay spoofing devices according to the regular hexagonal network type. For the target receiver with the robust adaptive clock difference jump detection function, the optimal value of the coverage radius of a single spoofing device is determined to be 10 km, and the optimal value of the distance between adjacent spoofing devices is determined to be 17 km [18]. Song Zhang et al. established the mathematical model of multi-objective planning and array optimization of the minimum and maximum evaluation function, and solved the model by the multiplier penalty function method. Under the condition of long-term operation of the system, based on the method of nonlinear programming, the motion model of the air platform was established, and the solution steps of the quadratic programming quadratic constraint motion model were given [19]. The main contribution of the above research results is to preliminarily explore the theoretical possibility of multiple spoofers to spoof together. However, first of all, there is a lack of specific analysis of the scope of forwarding spoofing; second, it fails to provide a reasonable deployment plan according to the specific location of victim receivers; and finally, no reasonable deployment scheme was designed according to the different spoofing requirements of the spoofer.

What is more, for multi-target navigation terminals that form a group with each other, such as unmanned aerial vehicles (UAVs), each UAV can resist spoofing by detecting the relative position. At present, the spoofer still lacks a timely and effective regional deployment algorithm to achieve collective spoofing. At the same time, the excellent regional deployment algorithm of distributed GNSS spoofers can provide a technical route for building a perfect GNSS spoofing defense zone. At this time, the spoofer does not excessively cling to the spoofing effect of a certain target but takes the overall spoofing success rate of multiple targets in the spoofing defense zone as an evaluation index. Therefore, it is extremely urgent to propose a regional deployment algorithm of distributed GNSS forwarding spoofing for multiple targets.

The structure and contents of this paper are as follows. The introduction summarizes the research background and current situation of distributed GNSS forwarding spoofing. In Section 1, the GNSS forwarding spoofing model is established, including the GNSS asynchronous traction spoofing method and the forwarding spoofing mathematical model. In Section 2, the scope of the forwarding spoofing is analyzed and evaluated. In Section 3, the forwarding spoofing region deployment algorithm for multi-spoofer multi-target (MSMT) is proposed. In Section 4, different MSMT spoofing scenarios are analyzed, and the performance of the proposed algorithm is verified and evaluated. Section 4 gives a comprehensive summary and a prospect of this paper.

## 2. GNSS Forwarding Spoofing Model

### 2.1. Mathematical Model of Forwarding Spoofing

**Figure 1:**
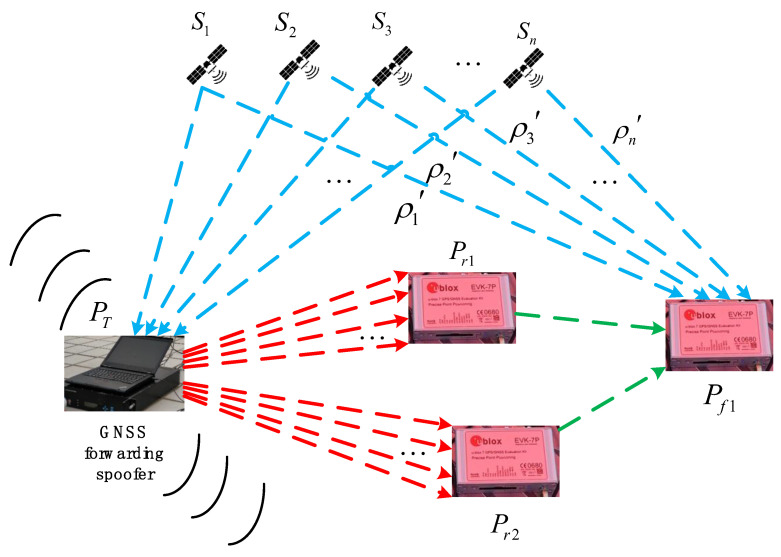
Mathematical model of forwarding spoofing.

The purpose of the forwarding spoofer is to spoof the positioning result of a victim receiver whose real physical location is $P_{r1}$ to the point $P_{f1}$. From the spoofer’s point of view, the forwarding spoofer first receives authentic signals from the sky, and the corresponding satellite positions are expressed as $S_{1}$, $S_{2}$, ⋯, and $S_{n}$. Then, according to the purpose of spoofing, the spoofer adds the corresponding time delay to each channel of authentic signals, and finally broadcasts through the omnidirectional antenna. As shown in Figure 1, here we aim at a more advanced forwarding spoofer; that is, the forwarding spoofer can separate different signals and add different time delays to spoof the receiver at point $P_{r1}$ to point $P_{f1}$.

On the one hand, the retransmitted signal does not change the navigation message. After demodulating the navigation message, the victim receiver will obtain the real satellite position information. The real positions of the n satellites used by the receiver for a positioning solution are respectively expressed as $S_{1}$, $S_{2}$, ⋯, and $S_{n}$. On the other hand, for the signals of these n satellites, the propagation time from satellite to spoofer and the processing delay are expressed as $t_{1}$, $t_{2}$, ⋯, and $t_{n}$, and the forwarding spoofer also performs corresponding delay processing, as shown in Figure 1. Therefore, the following expression is satisfied for a spoofing signal:

(1) $$A:\left\{ \begin{array}{l} \rho_{1}{}^{\prime }=c\cdot t_{1}+\left|P_{T}-P_{r1}\right|\\ \rho_{2}{}^{\prime }=c\cdot t_{2}+\left|P_{T}-P_{r1}\right|\\ \vdots \\ \rho_{n}{}^{\prime }=c\cdot t_{n}+\left|P_{T}-P_{r1}\right| \end{array}\right.$$

where $\rho_{1}{}^{\prime }$, $\rho_{2}{}^{\prime }$, ⋯, and $\rho_{n}{}^{\prime }$ represent the pseudorange measurements when the victim receiver receives spoofing signal for positioning, and c represents the speed of light. Meanwhile, when a victim receiver receives a spoofing signal for positioning, it satisfies the following equation:

(2) $$V:\left\{ \begin{array}{l} \left|S_{1}-P_{f1}\right|=\rho_{1}{}^{\prime }-c\cdot \delta t_{k}\\ \left|S_{2}-P_{f1}\right|=\rho_{2}{}^{\prime }-c\cdot \delta t_{k}\\ \vdots \\ \left|S_{n}-P_{f1}\right|=\rho_{n}{}^{\prime }-c\cdot \delta t_{k} \end{array}\right.$$

where $\delta t_{k}$ represents the receiver clock offset calculated by the victim receiver. Different from generative spoofing, since the delay added by the spoofer to each channel of signals is positive, the corresponding propagation times $t_{1}$, $t_{2}$, ⋯, and $t_{n}$ need to meet the following restrictions:

(3) $$A:\left\{ \begin{array}{l} c\cdot t_{1}>\left|S_{1}-P_{T}\right|\\ c\cdot t_{2}>\left|S_{2}-P_{T}\right|\\ \vdots \\ c\cdot t_{n}>\left|S_{n}-P_{T}\right| \end{array}\right.$$

It should be pointed out that for the multi-channel retransmitted signal broadcast by single omnidirectional antenna, if a spoofer keeps the above spoofing purpose unchanged, that is, the positioning result of victim receiver with the real physical position of $P_{r1}$ is spoofed to point $P_{f1}$, the spoofing signal can still spoof another victim receiver with the real physical position of $P_{r2}$, as shown in Figure 1. For the receiver at point $P_{r2}$, if a spoofing signal has a sufficient power advantage, the tracking loop can still be directly destroyed to control the positioning of the receiver. At this time, the spoofing signal satisfies the following equation:

(4) $$A:\left\{ \begin{array}{l} \rho_{1}{}^{\prime }=c\cdot t_{1}+\left|P_{T}-P_{r2}\right|\\ \rho_{2}{}^{\prime }=c\cdot t_{2}+\left|P_{T}-P_{r2}\right|\\ \vdots \\ \rho_{n}{}^{\prime }=c\cdot t_{n}+\left|P_{T}-P_{r2}\right| \end{array}\right.$$

Similarly, the propagation times $t_{1}$, $t_{2}$, ⋯, and $t_{n}$ at this time still need to satisfy the restriction condition of Equation (3). Meanwhile, when a victim receiver at $P_{r2}$ receives a spoofing signal for positioning, the following equation is satisfied:

(5) $$V:\left\{ \begin{array}{l} \left|S_{1}-P_{f1}\right|=\rho_{1}{}^{\prime }-\left(c\cdot \delta t_{k}+\left|P_{T}-P_{r2}\right|-\left|P_{T}-P_{r1}\right|\right)\\ \left|S_{2}-P_{f1}\right|=\rho_{2}{}^{\prime }-\left(c\cdot \delta t_{k}+\left|P_{T}-P_{r2}\right|-\left|P_{T}-P_{r1}\right|\right)\\ \vdots \\ \left|S_{n}-P_{f1}\right|=\rho_{n}{}^{\prime }-\left(c\cdot \delta t_{k}+\left|P_{T}-P_{r2}\right|-\left|P_{T}-P_{r1}\right|\right) \end{array}\right.$$

For the above equation, we need to explain that, according to the principle of single-point pseudorange positioning, the calculated receiver clock offset is the common deviation part [20] in the range observation of each satellite. Therefore, compared with the case of Equation (2), the increased common part $\left|P_{T}-P_{r2}\right|-\left|P_{T}-P_{r1}\right|$ of each pseudorange observation will be absorbed by receiver clock offset at this time, and the victim receiver will still be located at point $P_{f1}$, as shown in Figure 1. According to the above inference, it can be explained that if a spoofer wants to cheat the multiple targets constituting the group, on the one hand, different single targets will be spoofed to different positions and, at the same time, the relative positions between the single targets in the group will remain unchanged; then the spoofer cannot achieve this goal through a single forwarding spoofer. Therefore, it is necessary to consider deploying multiple spoofers in a distributed manner to jointly achieve the spoofing purpose.

### 2.2. GNSS Asynchronous Traction Spoofing Method

On the basis of Section 2.1, the process of invading the loop and controlling the loop after a retransmitted signal reaches the antenna of a victim receiver will be discussed below.

For asynchronous traction spoofing in forwarding spoofing, although the forwarding signal usually lags behind the authentic signal by a certain time delay, if the time delay is not too large, the correlation peaks formed by the forwarding signal and authentic signal at the tracking loop may overlap. In this case, the spoofing signal intrudes into the loop at a power higher than the authentic signal, and then, in the process of spoofing signal gradually moving the correlation peak, the loop can naturally lock to the higher power spoofing signal correlation peak through the power advantage.

When a GNSS retransmitted signal intrudes into the loop that remains locked to authentic satellite signal, taking the P branch of a loop as an example, its coherent integral output is expressed as [21]:

(6) $$P\left\{ \begin{array}{c} I_{P}\left(n\right)=\sqrt{P^{a}}D\left(n\right)R\left(\tau_{P}^{a}\right)\mathrm{sinc}(f_{e}^{a}T_{coh})cos(\phi_{e}^{a})\\ +\sqrt{P^{s}}D\left(n\right)R\left(\tau_{P}^{s}\right)\mathrm{sinc}(f_{e}^{s}T_{coh})cos(\phi_{e}^{s})\\ Q_{P}\left(n\right)=\sqrt{P^{a}}D\left(n\right)R\left(\tau_{P}^{a}\right)\mathrm{sinc}(f_{e}^{a}T_{coh})\sin(\phi_{e}^{a})\\ +\sqrt{P^{s}}D\left(n\right)R\left(\tau_{P}^{s}\right)\mathrm{sinc}(f_{e}^{s}T_{coh})sin(\phi_{e}^{s})\\ P\left(n\right)=\sqrt{I_{P}^{2}\left(n\right)+Q_{P}^{2}\left(n\right)} \end{array}\right.$$

where the symbol superscript s represents the spoofing signal, and the symbol superscript a represents the authentic signal. $I_{P}\left(n\right)$ and $Q_{P}\left(n\right)$ respectively, represent the output of the L branch and Q branch of the P channel. P represents the received signal power, D(n) represents the message bit level with value of ±1, $\tau_{P}$ represents the phase difference between copied C/A code and received C/A code of the P channel, d represents the distance between two adjacent correlators, and R(⋅) represents the code autocorrelation function. $f_{e}$ represents the frequency difference between local replica carrier and received signal carrier, $T_{coh}$ represents the coherent integration time, and $\phi_{e}$ represents the phase difference between local replica carrier and received signal carrier. P(n) represents the C/A code autocorrelation amplitude of the P branch [21]. The asynchronous traction spoofing process is further described in detail below.

**Figure 2:**
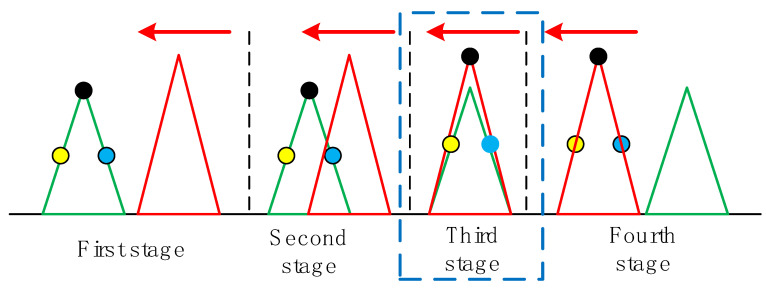
Asynchronous traction spoofing process.

The asynchronous traction spoofing process is shown in Figure 2. From left to right, there are four stages sorted by time. In the first stage, the receiver tracks the green authentic signal. The yellow dot, the black dot, and the blue dot represent the early, prompt, and late correlation points of the loop, respectively. The red spoofing signal has a certain power advantage over the authentic signal and lags behind the authentic signal by a certain time delay but is not far away. In the second stage, the spoofing signal gradually approaches the authentic signal, and the correlation peaks of the two signals overlap. In the third stage, the correlation peaks of the spoofing signal and authentic signal are basically aligned, and the spoofing signal has a power advantage over the authentic signal, thereby taking over the loop. In the fourth stage, the spoofing signal continues to move and manipulates the receiver to change the positioning result. In Figure 2, we have highlighted the third stage, which is the key step to realize spoofing by forwarding signals.

Another, perhaps more general, scenario is discussed below. That is, the retransmitted signal lags behind the authentic signal by a large time delay, and the correlation peaks of two signals basically do not overlap. At this time, the retransmitted signal will cause the tracking loop of the victim receiver to enter the state of losing lock and recapture through the high power. This is because the high-power retransmitted signal will bring stronger correlation noise, and the authentic signal will be submerged under the noise, so that the receiver will lose lock. Then the loop will capture and track the retransmitted signal with a large probability, thus realizing spoofing.

## 3. Evaluation of Scope of Action of Forwarding Spoofing

For a single forwarding spoofer that uses an omnidirectional antenna to broadcast spoofing signals, it is necessary to evaluate the spoofing effect of the area covered by its spoofing signals, which is the premise of designing a spoofing area deployment algorithm in the next section.

First, the situation in Section 2.1 is still used. The purpose of the spoofer is to spoof the positioning result of a victim receiver whose real physical location is $P_{r1}$ to $P_{f1}$. In general, at this time, for a victim receiver at $P_{r1}$ point, the spoofer can adjust the broadcast power of the spoofing signal by observing the position of the victim receiver, so that the power advantage η of the spoofing signal relative to the authentic signal is 30 dB. It should be noted that many research results show that when the power gain of a forwarding spoofing signal at the antenna of a victim receiver is more than 30 dB relative to an authentic signal, the receiver tracking loop will lose lock, and the recapture will capture and track the forwarding spoofing signal with a high probability [11,22]. Therefore, here, the spoofer uses a power gain of 30 dB as the minimum power gain value required to achieve spoofing. It should be noted that although the minimum power gain required by a spoofing signal to achieve spoofing will be different for different receivers with different configurations, the spoofing signal adopts a 30 dB power gain here for the convenience of analysis, which does not affect the final analysis result.

For a victim receiver at any point other than $P_{r1}$, take victim receiver at point $P_{r2}$ in the case of Section 2.1 as an example. According to Equation (5), at this time, compared with the case of a spoofing victim receiver at point $P_{r1}$, for victim receiver at point $P_{r2}$, the common increment $\left|P_{T}-P_{r2}\right|-\left|P_{T}-P_{r1}\right|$ of each pseudorange measurement of the spoofing signal is equivalent to the increased distance difference between the propagation delay of the spoofing signal and authentic signal. On the other hand, this will also cause free space propagation loss of GNSS signal power gain. Therefore, the influence of the code phase difference t between the spoofing signal and authentic signal and the power gain of the spoofing signal on the spoofing effect will be analyzed below.

Taking the typical commercial receiver loop configuration as an example, the distance between two adjacent correlators of the loop is d, where the common value is 0.5 chips. The loop adopts the incoherent lead-minus-lag power method, and the calculation equation of the unitary phase detection result $\delta_{cp}$ is Equation (7), where E and L represent the C/A code autocorrelation amplitudes of branch E and branch L, respectively. The pull in range of the loop is [−d,+d], i.e., 2d. When the code phase difference between received signal and the local signal exceeds ±d, the loop will lose lock.

(7) $$\delta_{cp}=\frac{E^{2}-L^{2}}{2\left(E^{2}+L^{2}\right)}$$

On the other hand, according to the free space propagation loss equation of GNSS signal, if the propagation distance is L and the signal wavelength is λ, the corresponding propagation loss is [20]:

(8) $$P_{s}=20\mathrm{lg}\left(\frac{\lambda }{4\pi L}\right)$$

**Figure 3:**
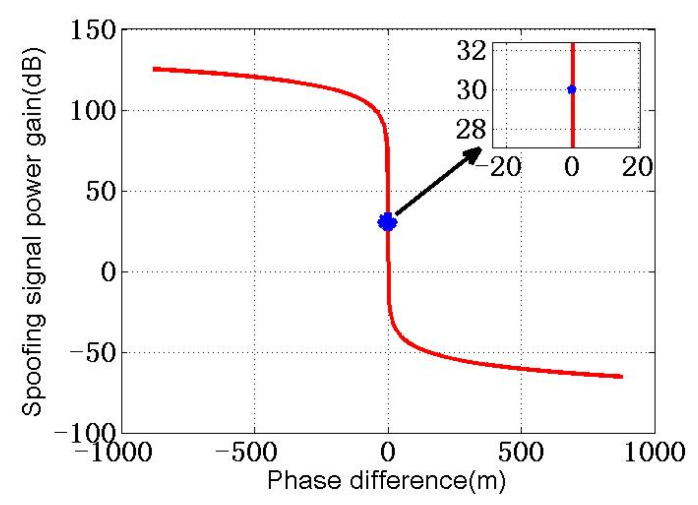
Power gain versus code phase difference.

When the forwarding spoofing signal reaches the receiver loop, when code phase difference between it and authentic signal is Δτ=D, D represents a negative value. The spoofing signal power gain is 30 dB. According to Equation (8), the relationship curve of spoofing signal power gain η with the code phase difference Δτ is shown in Figure 3.

**Figure 4:**
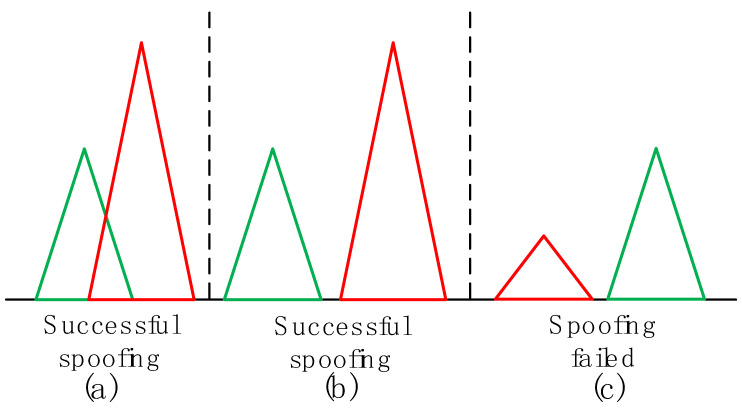
Schematic diagram of correlation peak between spoofing signal and authentic signal.

It should be noted that, for the convenience of representation, the abscissa in Figure 3 has normalized the code phase difference; that is, when the power gain of the spoofing signal is 30 dB, the code phase difference Δτ=D is represented by 0. According to Figure 3, when Δτ<D, the power gain η of the spoofing signal is much higher than 30 dB, while Δτ>D, the power gain η of the spoofing signal is much lower than 30 dB. To more vividly express the change of the correlation peak of the spoofing signal and authentic signal with the code phase difference, the schematic diagram of the correlation peak of the two signals is drawn as shown in Figure 4.

According to the above analysis, the following conclusions can be obtained:

(9) $$\left\{ \begin{array}{l} \Delta \tau =D\;,\;\eta =30\;,\;\mathrm{Successful}\;\mathrm{spoofing}\\ if\;\left(D<-4d\right)\;,\;D<\Delta \tau <-4d\;,\;\eta \gg 30\;,\;\mathrm{Successful}\;\mathrm{spoofing}\\ if\;\left(D<-4d\right)\;,-4d\leq \Delta \tau <0\;,\;\eta \gg 30\;,\;\mathrm{Successful}\;\mathrm{spoofing}\\ if\;\left(D>-4d\right)\;,-4d\leq \Delta \tau <D\;,\;\eta \ll 30\;,\;\mathrm{Spoofing}\;\mathrm{failed}\\ if\;\left(D>-4d\right)\;,\;\Delta \tau <-4d\;,\;\eta \ll 30\;,\;\mathrm{Spoofing}\;\mathrm{failed} \end{array}\right.$$

Equation (9) will be described below. When the retransmitted spoofing signal reaches a receiver loop, when the code phase difference between it and the authentic signal is Δτ=D, the power gain of the spoofing signal is 30 dB. At this time, forwarding spoofing can be realized. Since the receiver is in the critical state of losing lock, it can also be called critical spoofing; this is also critical power forwarding spoofing. However, there are two cases in which the spoofing signal lags behind the authentic signal delay; that is, the correlation peaks of the two still overlap and do not overlap, as shown in Figure 4a,b. 

The phase difference between the forwarding spoofing signal and authentic signal is usually less than 0, and when the phase difference is greater than −4d, the correlation peaks of the two will overlap. Therefore, if D<−4d, when code phase difference Δτ is between D and −4d, there is no overlap between the correlation peak of the spoofing signal and the correlation peak of the authentic signal; that is, the spoofing signal does not generate correlation interference with the authentic signal, but because the power gain η of the spoofing signal is much higher than 30 dB, the loop can be unlocked and the spoofing signal can be recaptured, and the spoofing is successful, that is, the situation in Figure 4b.

If D<−4d, when code phase difference Δτ is between −4d and 0, the correlation peak of spoofing signal and correlation peak of authentic signal still overlap; that is, the spoofing signal can generate correlation interference to the authentic signal, and the power gain η of the spoofing signal is much higher than 30 dB, and then the loop can be unlocked and spoofing signal can be recaptured, and spoofing is successful, that is, the situation in Figure 4a.

If D>−4d, when code phase difference Δτ is between −4d and D, the correlation peak of spoofing signal and correlation peak of authentic signal still overlap; that is, the spoofing signal can generate correlation interference to the authentic signal, but the power gain η of spoofing signal is much lower than 30 dB, and then spoofing fails, that is, the situation in Figure 4c.

If D>−4d, when code phase difference Δτ is less than −4d, there is no overlap between the correlation peak of the spoofing signal and correlation peak of the authentic signal; that is, the spoofing signal does not generate correlation interference with the authentic signal, and the power gain η of the spoofing signal is much lower than 30 dB, and then spoofing fails, that is, the situation in Figure 4c.

**Figure 5:**
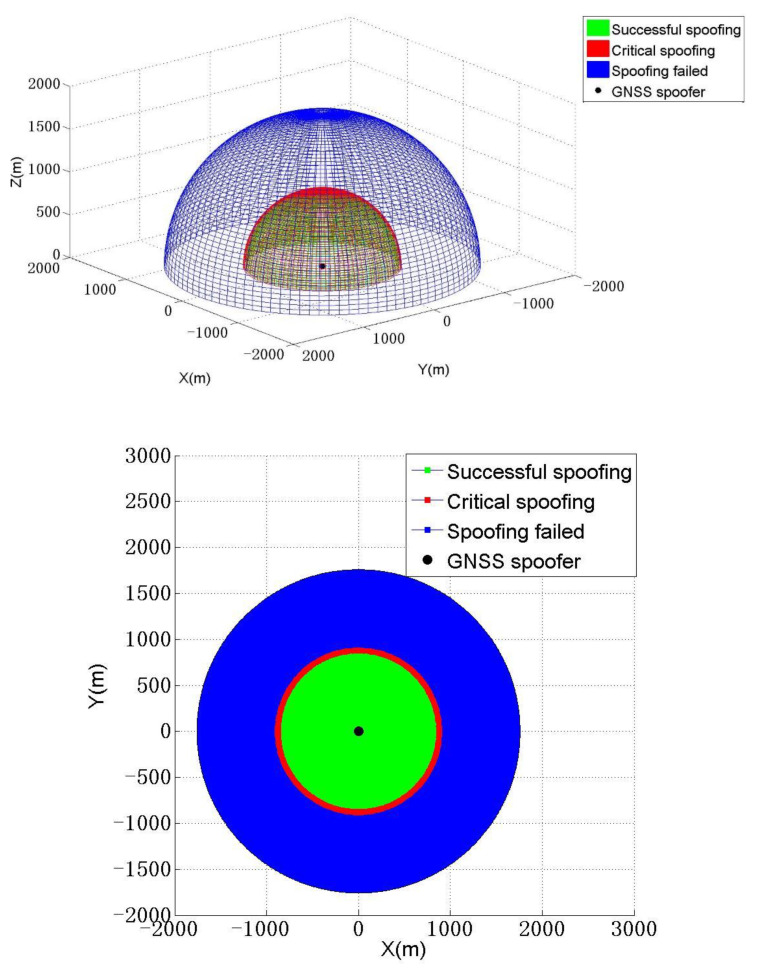
**Three-dimensional** schematic diagram (**top**) and two-dimensional schematic diagram (**bottom**) of action scope of different spoofing effects.

According to the above analysis, it is possible to obtain the schematic diagram of the action range of different spoofing effects of a forwarding spoofing device that uses an omnidirectional antenna to broadcast spoofing signals. The three-dimensional and two-dimensional plane schematic diagrams are shown in Figure 5(top), (bottom), respectively.

As shown in Figure 5(top), when a victim receiver is located on the red surface, the spoofer can realize successful critical spoofing on it and define the red surface position as a critical spoofing area. When a victim receiver is located in the green area, the spoofer can successfully cheat it. When a victim receiver is located in the blue area, the spoofer cannot realize any kind of spoofing.

As shown in Figure 5(bottom), when a victim receiver is located on the red line, the spoofer can realize successful critical spoofing on it and define the red line position as a critical spoofing area. When a victim receiver is located in the green area, the spoofer can successfully cheat it. When a victim receiver is located in the blue area, the spoofer cannot cheat it.

## 4. Forwarding Spoofer Area Deployment Algorithm for MSMT

According to the spoofing purpose of a spoofer to the group of multiple receivers, we propose two forwarding spoofer area deployment algorithms for MSMT scenarios. The first is the multi-target same-point spoofing algorithm (MSPSA), and the second is the multi-target different-point spoofing algorithm (MDPSA). The scenarios are all conducted in a two-dimensional plane. In actual spoofing, it is impossible for the spoofer to be infinitely far away from receivers. It is assumed that the possible deployment location of a spoofer is also limited; that is, we demarcate all spoofers and receivers within a given geometric area RE, which is also a given area of the spoofing scene. At this time, the deployment location of a spoofer can only be selected within a given area.

### 4.1. MSPSA for Group Multiple Receivers

In this scenario, a spoofer expects to spoof the positioning results of six receivers located at different positions in the group to the same spoofing point $P_{f1}$. We define this situation as “same-point spoofing”. To achieve the purpose of spoofing, according to Section 2.1, at this time, a spoofer can cheat multiple receivers at the same time through a forwarding spoofer. The MSPSA applicable to this scenario will be described in detail below.

Step 1: it is assumed that a spoofer obtains the positions of the six victim receivers in the group by radar observation, which are expressed as $P_{r1}$, $P_{r2}$, ⋯, and $P_{r6}$.

Step 2: a unique circle can be determined according to the three noncollinear points on the plane. Among the six victim receivers, any three receivers located in noncollinear positions $P_{r1}$, $P_{r2}$, and $P_{r3}$ can be selected. Taking these three receivers as points on the circle, a unique circle can be formed. According to the principle of permutation and combination, if any three receivers in the group are not collinear, there will be $W=C_{6}^{3}=\frac{6!}{3!\left(6-3\right)!}=20$ combinations.

Take one of the combinations, i, as an example. It is assumed that in combination i, three receivers $P_{r1}$, $P_{r2}$, and $P_{r3}$ form a circle, which is defined as the forwarding spoofing circle $C_{1}$. In fact, this is a forwarding spoofing circle formed by a single GNSS forwarding spoofing device deployed in $P_{T1}$, as shown in Figure 5(bottom). $P_{T1}$ is also the center position of the forwarding spoofing circle $C_{1}$. The victim receivers located on the circle will be subjected to critical spoofing, while receivers located inside the circle will be subjected to higher-power forwarding spoofing. To improve the concealment of spoofing, the spoofer obviously prefers that victim receivers are all located at the critical spoofing position because this requires the minimum power to realize forwarding spoofing; it is considered here that forwarding signals will hardly affect the receiver located outside the circle.

Step 3: calculate the positional relationship between the remaining three receivers located at $P_{r4}$, $P_{r5}$, and $P_{r6}$ and the spoofing circle $C_{1}$. If one of the remaining three receivers is located on the spoofing circle $C_{1}$, it will be subjected to critical spoofing by the spoofing device located at $P_{T1}$. If there is one located within spoofing circle $C_{1}$, it will be spoofed by higher-power forwarding spoofing by a spoofer located at $P_{T1}$. If there is one outside spoofing circle $C_{1}$, it will not be affected by the spoofer at $P_{T1}$.

Step 4: according to the judgment in step 3, there will be the following four situations.

**Figure 6:**
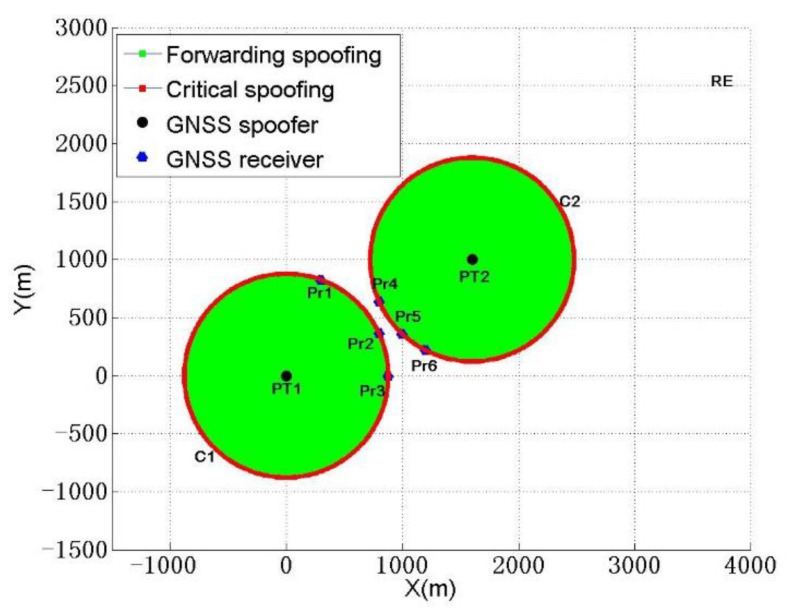
Schematic diagram of forwarding spoofer deployment in MSPSA.

(1) Case 1: among the remaining three receivers, three are outside spoofing circle $C_{1}$. The unique spoofing circle $C_{2}$ is formed by three noncollinear receivers, $P_{r4}$, $P_{r5}$, and $P_{r6}$, other than spoofing circle $C_{1}$, which is formed by a single GNSS forwarding spoofing device deployed in $P_{T2}$, as shown in Figure 6.

Subsequently, it is determined that the three receivers located at $P_{r1}$, $P_{r2}$, and $P_{r3}$, other than the receivers located at $P_{r4}$, $P_{r5}$, and $P_{r6}$, intersect with spoofing circle $C_{1}$ and spoofing circle $C_{2}$. The equation is expressed as (10); $d_{2i}$ and $d_{1i}$, respectively, represent the distances from the center $\left(x_{2},\;y_{2}\right)$, $\left(x_{1},\;y_{1}\right)$ of the corresponding spoofing circle to the ith receiver. When the receiver is located on spoofing circle $C_{2}$, the spoofing fails because of signal crosstalk with spoofing circle $C_{1}$. When the receiver is located inside spoofing circle $C_{2}$, spoofing fails in the same way. When the receiver is outside spoofing circle $C_{2}$ and on spoofing circle $C_{1}$, the receiver is critically spoofed. When the receiver is outside spoofing circle $C_{2}$ and within spoofing circle $C_{1}$, the receiver is spoofed by higher-power forwarding spoofing.

(10) $$\left\{ \begin{array}{l} d_{2i}=R_{2}\;,\;\mathrm{Spoofing}\;\mathrm{failed}\\ d_{2i}<R_{2}\;,\;\mathrm{Spoofing}\;\mathrm{failed}\\ \left(d_{2i}>R_{2}\right)\&\&\left(d_{1i}=R_{1}\right)\;,\;\mathrm{Critical}\;\mathrm{spoofing}\\ \left(d_{2i}>R_{2}\right)\&\&\left(d_{1i}<R_{1}\right)\;,\;\mathrm{Higher}\;\mathrm{power}\;\mathrm{forwarding}\;\mathrm{spoofing} \end{array}\right.$$

Thus, the deployment positions of the two spoofers are determined, and the spoofing situations of the receivers are obtained.

(2) Case 2: two of the remaining three receivers are outside spoofing circle $C_{1}$. ① When the two receivers $P_{r5}$ and $P_{r6}$ form a spoofing circle, spoofing circles $C_{2}$ and $C_{3}$ pass through the two receivers respectively.

Spoofing circle $C_{2}$ is determined by the optimal programming algorithm. First, the center $\left(x_{2},\;y_{2}\right)$ of spoofing circle $C_{2}$ is on the perpendicular line of line segment $P_{r5}P_{r6}$. Furthermore, to pursue the concealment of a spoofer in the geographical position, the shortest sum of the distances between the center $\left(x_{2},\;y_{2}\right)$ of spoofing circle $C_{2}$ and all six receivers is taken as the objective function. At the same time, there are two constraints: the first is that the other six receivers cannot intersect with spoofing circle $C_{2}$, and the second is that spoofing circles $C_{2}$ and $C_{1}$ cannot intersect, as shown in Equation (11).

(11) $$\left\{ \begin{array}{l} \min\;f\left(x_{2},\;y_{2}\right)=d_{21}+d_{22}+d_{23}+d_{24}+d_{25}+d_{26}\\ s.t.\;\left\{ \begin{array}{l} R_{2}<d_{31},R_{2}<d_{32},R_{2}<d_{33},R_{2}<d_{34}\\ R_{1}+R_{2}<D_{12} \end{array}\right. \end{array}\right.$$

According to Equation (11), the spoofing circle $C_{2}$ can be determined by the optimization programming algorithm.

(3) Case 3: among the remaining three receivers, one receiver located at $P_{r6}$ is outside spoofing circle $C_{1}$. Spoofing circle $C_{2}$ is determined by the optimal programming algorithm. To pursue the concealment of spoofing in the geographical position, the shortest sum of the distances between the center $\left(x_{2},\;y_{2}\right)$ of spoofing circle $C_{2}$ and all six receivers is taken as the objective function; At the same time, there are two constraints: the first is that the other five receivers cannot intersect with spoofing circle $C_{2}$, and the second is that spoofing circles $C_{2}$ and $C_{1}$ cannot intersect, as shown in Equation (12).

(12) $$\left\{ \begin{array}{l} \min\;f\left(x_{2},\;y_{2}\right)=d_{21}+d_{22}+d_{23}+d_{24}+d_{25}+d_{26}\\ s.t.\;\left\{ \begin{array}{l} R_{2}<d_{31},R_{2}<d_{32},R_{2}<d_{33},R_{2}<d_{34},R_{2}<d_{35}\\ R_{1}+R_{2}<D_{12} \end{array}\right. \end{array}\right.$$

According to Equation (12), spoofing circle $C_{2}$ can be determined by the optimization programming algorithm.

(4) Case 4: among the remaining three receivers, 0 is outside spoofing circle $C_{1}$. In this case, there is only one spoofer, and the relationship between the six victim receivers and spoofing circle $C_{1}$ is clear.

Step 5: steps 2–4 are all about combination i. At this time, all combinations are traversed. After all the above combinations are processed, the following four indexes, SN, $\sum d_{nm}^{Tr}$, CSR, and SSR, are proposed as the performance criteria for judging a spoofer deployment scheme:

(1) SN: number of GNSS forwarding spoofers used;
(2) $\sum d_{nm}^{Tr}$: the sum of the distances from each spoofer to each victim receiver;
(3) CSR: critical spoofing rate (CSR), the proportion of critical spoofed receiver to the number of all spoofed receivers;
(4) SSR: spoofing success rate (SSR), the proportion of spoofed receivers in the total number of receivers.

In MSPSA, the above four indexes will be calculated.

It is easy to understand that from the perspective of the equipment costs of a spoofer, it is expected that the less the index 1, the better. From the concealment of spoofing in geographical space, index 2 is the sum of all elements of $D_{Tr}$ in (13), where $d_{nm}^{Tr}$ represents the straight-line distance from the nth spoofing device to the mth victim receiver, and the larger the better. Index 3 is used to evaluate the concealment degree of spoofing performed by spoofer. The higher the CSR is, the more the spoofed receivers are subjected to critical spoofing. Index 4 is used to evaluate the success rate of spoofing task completed by spoofing device. The higher the SSR, the more receivers are successfully spoofed.

(13) $$D_{Tr}=\left[\begin{array}{ccccc} d_{11}^{Tr} & \cdots & d_{1m}^{Tr} & \cdots & d_{1n}^{Tr}\\ \vdots & \ddots & \vdots & \ddots & \vdots \\ d_{m1}^{Tr} & \cdots & d_{mm}^{Tr} & \cdots & d_{mn}^{Tr}\\ \vdots & \ddots & \vdots & \ddots & \vdots \\ d_{n1}^{Tr} & \cdots & d_{nm}^{Tr} & \cdots & d_{nn}^{Tr} \end{array}\right]$$

However, the importance of the four indexes will not be ranked here, which will be determined by the intention of spoofer. This means that spoofers will decide which deployment scheme to choose according to their specific actual needs, and MSPSA will provide all possible deployment schemes and corresponding indexes for a spoofer to choose.

**Figure 7:**
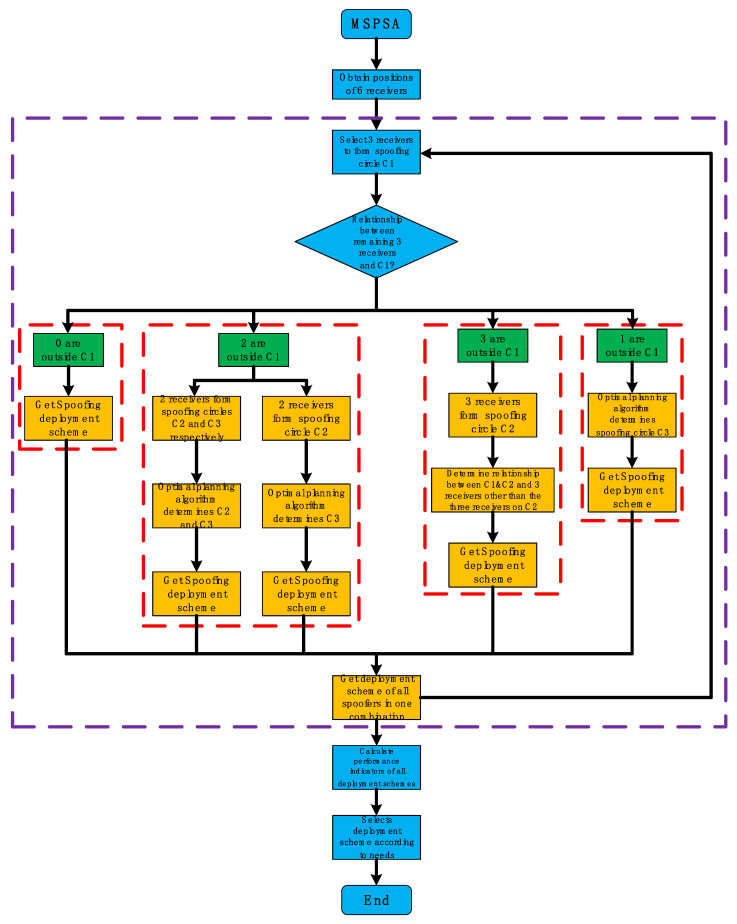
MSPSA execution flow chart.

According to the above detailed description of the algorithm steps, the key flow of MSPSA is given, as shown in Figure 7.

### 4.2. MDPSA for Group Multiple Receivers

In this scenario, a spoofer expects to spoof the positioning results of six victim receivers located at different positions $P_{r1}$, $P_{r2}$, ⋯, and $P_{r6}$ in the group to different spoofing points $P_{f1}$, $P_{f2}$, ⋯, and $P_{f6}$, while maintaining the relative positional relationship between the real positions of the receivers among these spoofing points. This situation is defined as “different-point spoofing”. To achieve the purpose of spoofing, according to Section 2.1, at this time, a spoofer needs to deploy six GNSS forwarding spoofing devices to spoof multiple receivers at the same time; that is, a single spoofing device only completes the spoofing of one receiver because this is “different-point spoofing”. The regional deployment algorithm applicable to this scenario will be described in detail below.

Step 1: it is assumed that a spoofer obtains the positions of the six victim receivers in the group by radar observation, which are expressed as $P_{r1}$, $P_{r2}$, ⋯, and $P_{r6}$.

**Figure 8:**
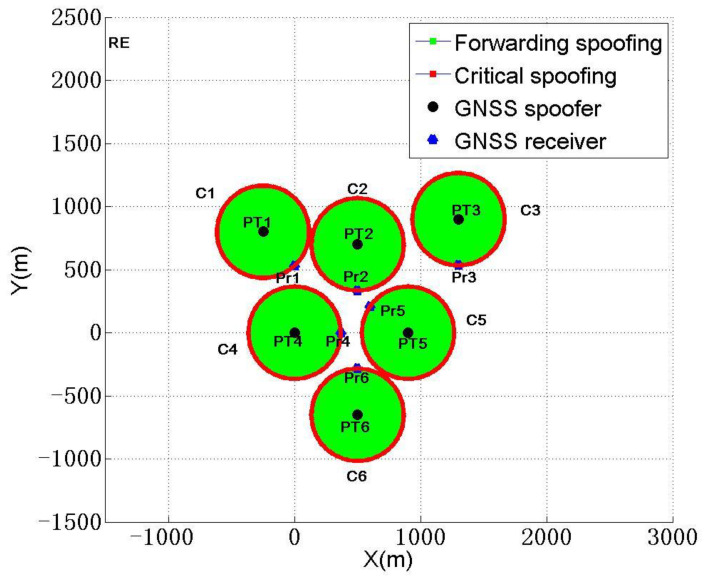
Schematic diagram of forwarding spoofer deployment in MDPSA.

Step 2: each spoofer spoofs one receiver, corresponding to the receiver located at $P_{r1}$. A single GNSS forwarding spoofer deployed at $P_{T1}$ forms forwarding spoofing circle $C_{1}$, as shown in Figure 5(bottom). $P_{T1}$ is the center position of forwarding spoofing circle $C_{1}$. The receiver located at $P_{r1}$ will be subjected to critical spoofing. Similarly, the receiver located inside the circle will be subjected to higher-power forwarding spoofing. The receiver outside circle will not be affected by a signal broadcast by a spoofer. Therefore, corresponding to the six victim receivers, the six spoofers will form six forwarding spoofing circles, $C_{1}$, $C_{2}$, ⋯, and $C_{6}$, as shown in Figure 8.

Step 3: the restriction conditions of six forwarding spoofing circles need to be considered. On the one hand, for any one of the forwarding spoofing circles $C_{m}$, except for the receiver located in $P_{rm}$ that it is responsible for spoofing, no victim receiver in other areas can intersect with forwarding spoofing circle $C_{m}$; otherwise, it will obviously cause unnecessary signal collision to the spoofing of other receivers and lead to possible spoofing failure. On the other hand, all spoofers are deployed in a given area RE, as shown in Equation (14).

(14) $$\left\{ \begin{array}{l} P_{r1}\in C_{1},\;P_{r2},P_{r3},\cdots ,P_{r6}⊄C_{1}\\ P_{r2}\in C_{2},\;P_{r1},P_{r3},P_{r4},\cdots ,P_{r6}⊄C_{2}\\ \cdots \\ P_{r6}\in C_{6},\;P_{r1},P_{r2},\cdots ,P_{r5}⊄C_{6}\\ P_{T1},P_{T2},\cdots ,P_{T6}\subseteq RE \end{array}\right.$$

Step 4: after the above restrictions are given, we still take the SN, $\sum d_{nm}^{Tr}$, CSR, and SSR proposed in MSPSA as the performance criteria for evaluating the spoofing deployment scheme. However, it should be noted that SN = 6, CSR = 100%, and SSR = 100% are easy to achieve in different-point spoofing. Therefore, we take index $\sum d_{nm}^{Tr}$ as the objective function and take (15) as the constraint condition to optimize the solution.

According to the above detailed description of the algorithm steps, we give the key processes of MDPSA, as shown in Table 1.

## 5. Experimental Analysis of Different MSMT Spoofing Scenarios

To fully verify and evaluate the performance of forwarding spoofer area deployment algorithms MSPSA and MDPSA for MSMT, the same-point spoofing experiment and different-point spoofing experiment on multiple receivers in the group are carried out in Section 5.1 and Section 5.2, respectively. The experiment is carried out on a self-developed spoofing software platform based on MATLAB. In the software platform, if the same-point spoofing or different-point spoofing is selected according to the two-dimensional coordinates of the victim receiver input, the MSPSA algorithm and the MDPSA algorithm are executed respectively. All mathematical models of these two algorithms are introduced in detail in Section 4.1 and Section 4.2 for repeatable research. After the implementation of the algorithm, the software finally gives the best deployment scheme of the spoofer according to the spoofer’s spoofing purpose and each scheme’s indexes.

### 5.1. Same Point Spoofing Experiments on Multiple Receivers in Group

In the experimental scenario, the spoofer intends to spoof all six receivers to the same spoofing point $P_{f1}$, and the plane coordinates of $P_{f1}$ are (1018, 1015). The spoofer obtains the positions of six victim receivers in the group $P_{r1}$, $P_{r2}$, ⋯, $P_{r6}$, and the corresponding plane coordinates are shown in Table 2.

After implementing the MSPSA algorithm proposed in Section 4.1, 25 deployment schemes will be given. Next, according to the different needs of the spoofer, the most suitable deployment scheme of the spoofer is obtained through analysis and comparison.

(1) When the spoofer expects to use the least number of spoofing devices to complete spoofing, there will be the following two schemes. The specific schemes and their performance indexes are shown in Table 3.

**Figure 9:**
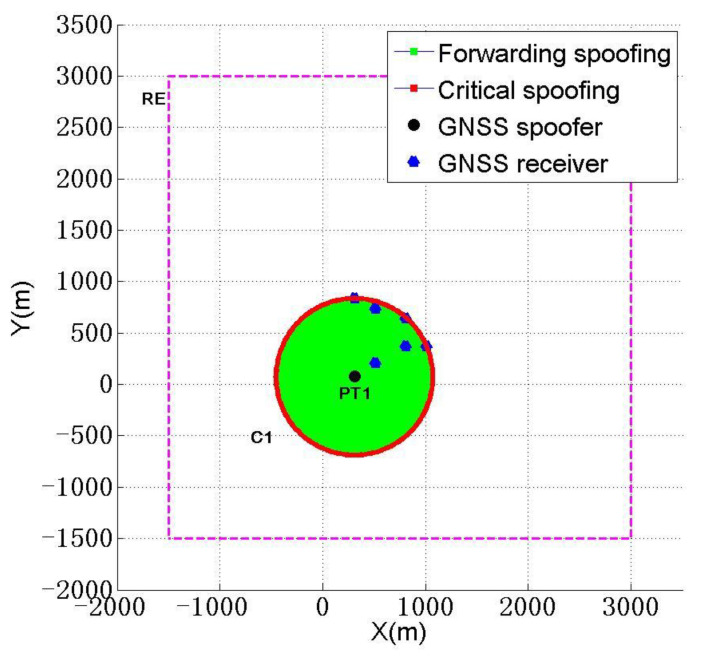
Deployment diagram of spoofers with the least-spoofing device.

According to Table 3, when the number of spoofers is one, compared with the above two schemes, the total distance $\sum d_{nm}^{Tr}$ between the spoofer and each receiver in scheme 2 is larger, $\sum d_{nm}^{Tr}=3815.6\;m$, and the deployment diagram of scheme 2 is shown in Figure 9.

RE refers to the allowable deployment range of the spoofer mentioned at the beginning of Section 4. Therefore, when a spoofer expects to use the least number of spoofing devices to complete spoofing, we think it is best to choose scheme2 in Table 3.

(2) When a spoofer expects the total distance $\sum d_{nm}^{Tr}$ between each spoofer and each receiver to be the largest, we list all schemes with $\sum d_{nm}^{Tr}>21\;\mathrm{km}$ and their performance indexes in Table 4.

**Figure 10:**
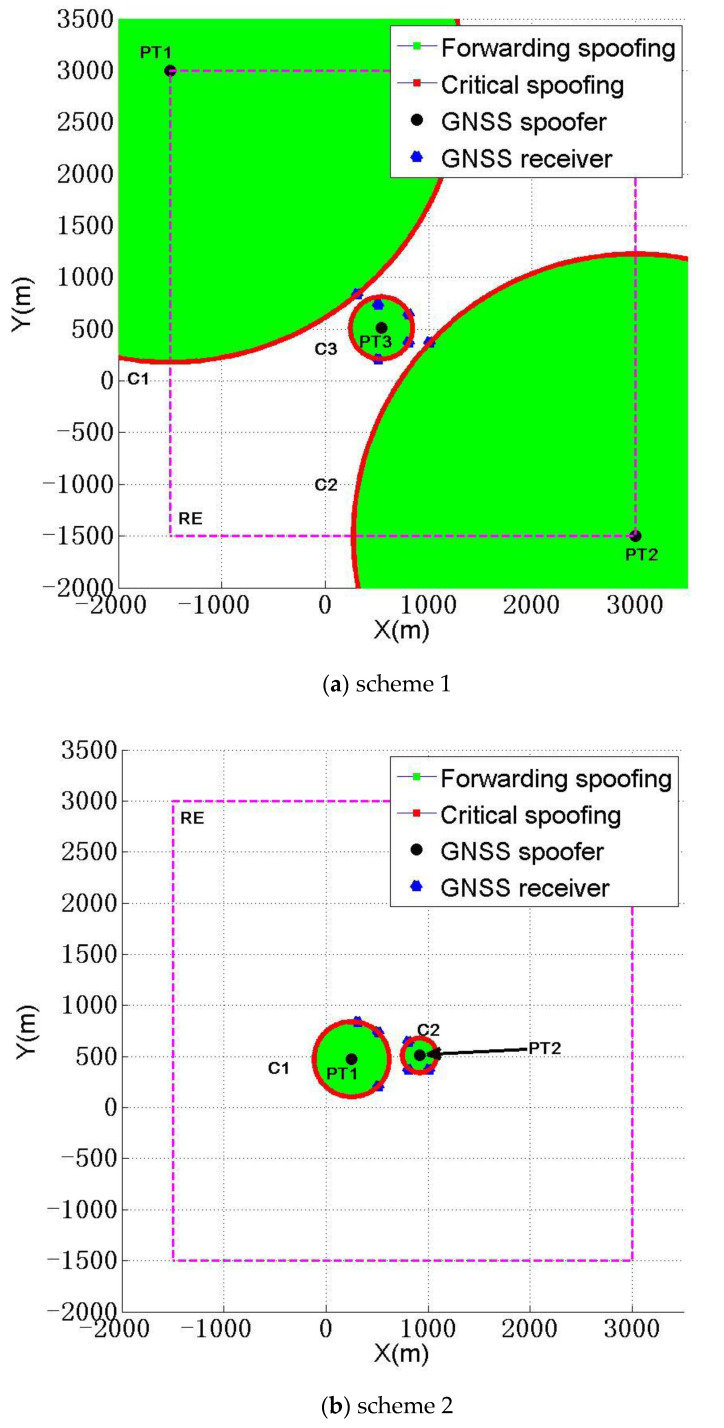
Deployment diagram of spoofers with the largest distance sum.

The eight schemes in Table 4 have the largest $\sum d_{nm}^{Tr}$ among all 25 schemes. Although a spoofer mainly pursues $\sum d_{nm}^{Tr}$ max at this time, it should be noted that the number of spoofing devices used in schemes 7 and 8 in Table 4 is 2, and the cost of the spoofing devices is lower than that of schemes 1 to 6. However, at the same time, the CSR of scheme 7 is reduced to 66.7%, that is, concealment of spoofing becomes worse. Although the CSR of scheme 8 is still 83.3%, but $\sum d_{nm}^{Tr}=21,928.1\;m$, compared with schemes 1 and 2, the $\sum d_{nm}^{Tr}$ of scheme 8 is significantly reduced, which makes it difficult to meet the requirements of the spoofer. The deployment diagrams of schemes 1 and 2 are shown in Figure 10,a,b.

Figure 10a,b show that three spoofers spoofed six receivers, five of which suffered critical spoofing and one of which suffered higher power forwarding spoofing.

Therefore, when a spoofer expects the sum $\sum d_{nm}^{Tr}$ of the distances between each spoofer and each receiver to be the largest, we think that it is best to select schemes 1 and 2 in Table 4.

(3) When a spoofer pursues the max of CSR and SSR at the same time, the number of spoofing devices are kept at two through statistics. Therefore, we list the deployment scheme and its performance indexes that satisfy CSR = 100% and SSR = 100% in Table 5.

**Figure 11:**
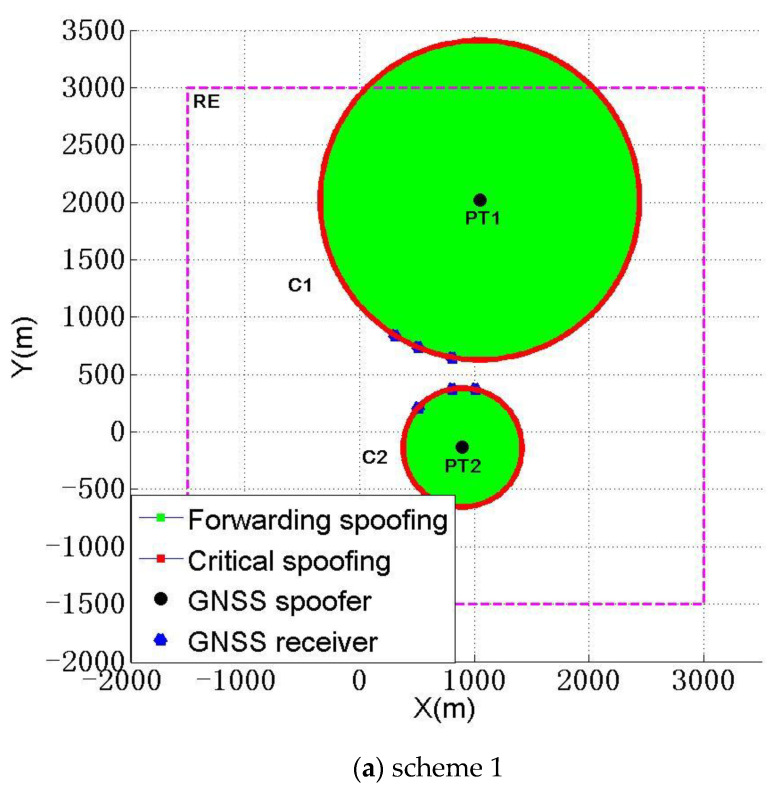

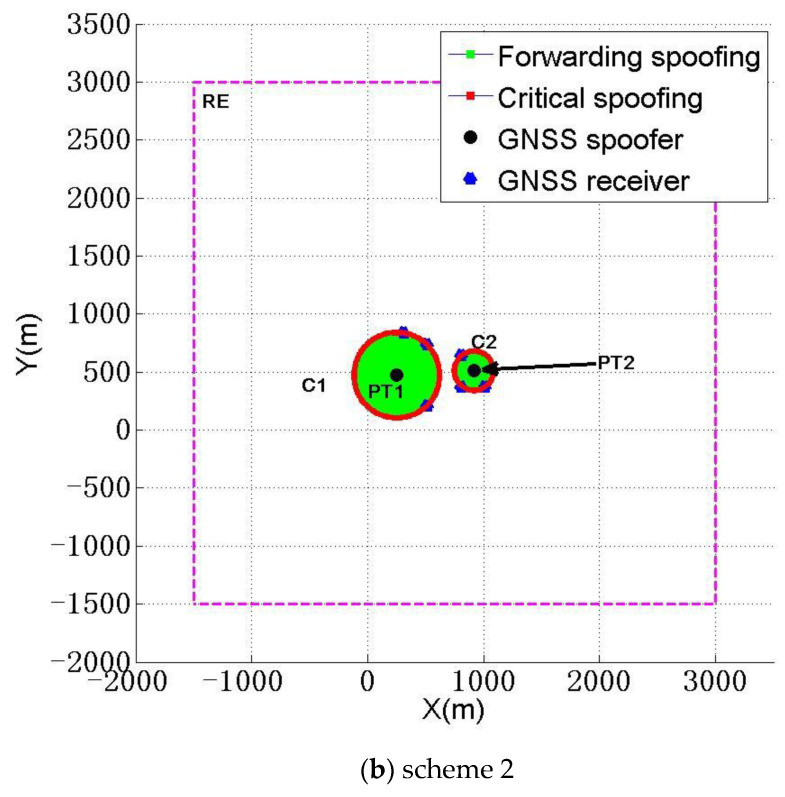
Deployment diagram of spoofers with the largest CSR and SSR.

The two schemes in Table 5 ensure that the CSR and SSR are 100% in all 25 schemes (in this case, the number of spoofers must be two). On the basis of meeting the needs of a spoofer, we consider ensuring that $\sum d_{nm}^{Tr}$ reaches the maximum. Obviously, scheme 1 and scheme 2 in Table 5 reach the max $\sum d_{nm}^{Tr}=13,803.5\;m$ and the sub max $\sum d_{nm}^{Tr}=5199.7\;m$ of $\sum d_{nm}^{Tr}$, respectively. The deployment of scheme 1 and scheme 2 are shown in Figure 11a,b.

Therefore, when a spoofer pursues the max CSR and SSR at the same time, we think it is very appropriate to choose scheme 1 and scheme 2 in Table 5.

### 5.2. Different-Point Spoofing Experiments on Multiple Receivers in Group

In the experimental scenario, the spoofer intends to spoof six receivers to different spoofing points $P_{f1}$, $P_{f2}$, ⋯, and $P_{f5}$. The spoofer obtains the positions of the six victim receivers in the group, $P_{r1}$, $P_{r2}$, ⋯, and $P_{r5}$, and the corresponding plane coordinates are shown in Table 6.

After executing the MDPSA algorithm proposed in Section 4.2, the following deployment scheme is given. The plane coordinates and action radius of each spoofer are shown in Table 7.

The performance indexes of the deployment scheme are shown in Table 8.

**Figure 12:**
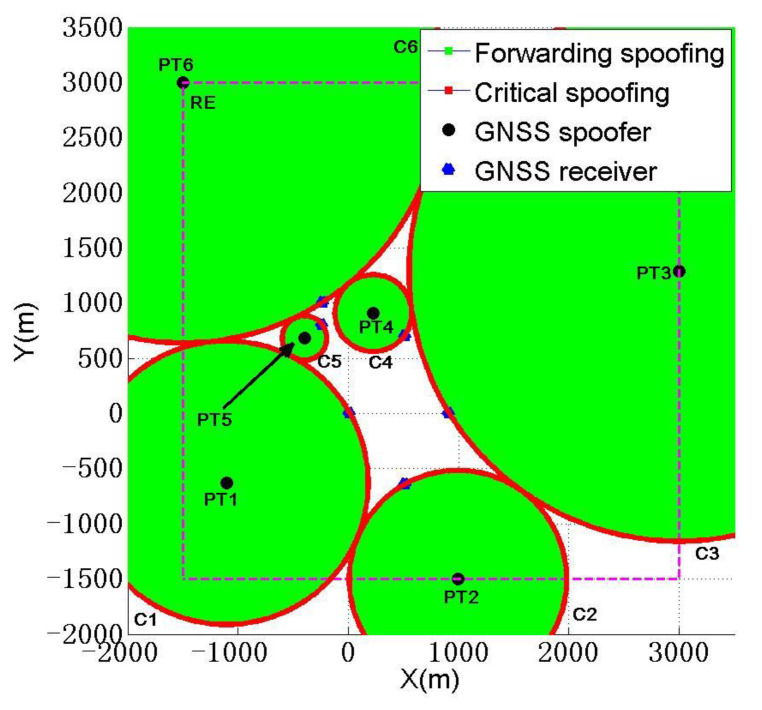
Schematic diagram of spoofing device deployment for different spoofings.

The deployment diagram of scheme 1 is shown in Figure 12.

According to the scheme in Table 7, six spoofing devices will be deployed at this time, all six receivers will be subjected to critical spoofing, and the CSR and SSR will reach 100%. At this time, $\sum d_{nm}^{Tr}$ will reach the max of 70,106.4 m, so this will be the best spoofing deployment scheme given.

In our MSPSA and MDPSA algorithms, for the same-point spoofing, when the spoofer expects to use the least spoofer to complete spoofing, one spoofer can complete spoofing, and the total distance between the spoofer and the receiver is 3815.6 m, and CSR and SSR are 50% and 100%, respectively. When a spoofer expects the maximum total distance between the spoofer and the receiver, five spoofers can be used to complete spoofing, and the total distance between the spoofer and the receiver is 40,345.1 m, and CSR and SSR are 83.3% and 100%, respectively. When the spoofer expects the CSR and SSR to be the maximum together, six spoofers can be used to complete spoofing. The total distance between the spoofer and the receiver is 13,803.5 m, and the CSR and SSR are 100% and 100%, respectively.

For the different-point spoofing, the deceiver uses six cheaters to complete the deception, and the total distance between the cheater and the receiver will reach the maximum of 70,106.4 m, with CSR and SSR of 100% and 100%, respectively.

The algorithm we proposed is based on the scope of the forwarding spoofing and realizes same-point spoofing and different-point spoofing. According to the different purposes of the spoofer, such as the minimum number of spoofers, the maximum distance between the spoofer and the receiver, and the maximum CSR and SSR together, the optimal spoofer deployment scheme is given. 

## 6. Conclusions and Future Work

To realize effective distributed forwarding spoofing for multiple receiver sensors in the area, we have done some useful research work. (1) The research status and key problems of distributed GNSS forwarding spoofing are summarized; (2) the forwarding spoofing model of GNSS is established, including a forwarding spoofing mathematical model and asynchronous traction spoofing method; (3) the scope of action of forwarding spoofing is evaluated in detail, and the schematic diagram of the scope of action of different spoofing effects is given; (4) two forwarding spoofer area deployment algorithms for MSMT are proposed—MSPSA is suitable for the same-point spoofing of multiple receiver sensors, and MDPSA is suitable for the different-point spoofing of multiple receiver sensors; and (5) the experimental tests of MSPSA and MDPSA for MSMT are carried out. The experimental results show that MSPSA and MDPSA can give the most appropriate deployment scheme of spoofing according to the different spoofing needs of spoofers, and the performance of the deployment scheme can meet the needs of spoofers.

Future work will be carried out from the following three aspects: (1) when the number of receivers in the area is larger and the types are different, it is necessary to explore more effective distributed spoofer deployment algorithms; (2) the area where the receiver is deployed may be at low altitude, which will bring many challenges to the deployment of spoofers—it is necessary to research the distributed spoofer deployment algorithm under low altitude conditions; and (3) when multiple receiver sensors in the area have certain anti-spoofing techniques, the spoofing strategy of a spoofing device needs to be adjusted flexibly, and the distributed spoofing device deployment algorithm for multiple anti-spoofing receivers needs to be explored.

## Figures and Tables

**Table 1:** Process of MDPSA.

MDPSA Different-Point Spoofing Algorithm for Group of Multiple Receivers
Input positions of 6 victim receivers: $P_{r1}$, $P_{r2}$, ⋯, $P_{r6}$
Form 6 forwarding spoofing circles: $C_{1}$, $C_{2}$, ⋯, $C_{i}$, ⋯, $C_{6}$$C_{i}:{\left(x-x_{P_{Ti}}\right)}^{2}+{\left(y-y_{P_{Ti}}\right)}^{2}={\left|P_{Ti}-P_{ri}\right|}^{2}$
Satify the constraints:
$\left\{ \begin{array}{l} P_{r1}\in C_{1},\;P_{r2},P_{r3},\cdots ,P_{r6}⊄C_{1}\\ P_{r2}\in C_{2},\;P_{r1},P_{r3},P_{r4},\cdots ,P_{r6}⊄C_{2}\\ \cdots \\ P_{r6}\in C_{6},\;P_{r1},P_{r2},\cdots ,P_{r5}⊄C_{6}\\ P_{T1},P_{T2},\cdots ,P_{T6}\subseteq RE \end{array}\right.$
Objective $\max\left(\sum d_{nm}^{Tr}\right)$, execute optimal algorithmCalculate 4 indexes SN, $\sum d_{nm}^{Tr}$, CSR, SSR
Get the best deployment scheme of spoofing

**Table 2:** Two-dimensional coordinates of victim receivers in MSPSA.

Victim Receiver	X (m)	Y (m)
$P_{r1}$	812	376
$P_{r2}$	512	735
$P_{r3}$	312	838
$P_{r4}$	512	212
$P_{r5}$	1012	370
$P_{r6}$	812	648

**Table 3:** Deployment scheme and various performance indexes when expect least spoofing devices.

Scheme Number	SR	Number of Critical Spoofed Receivers	Number of Higher Power Forwarding Spoofed Receivers	$\sum d_{nm}^{Tr}\;(m)$	CSR	SSR
1	1	3	3	1890.8	50%	100%
2	1	3	3	3815.6	50%	100%

**Table 4:** Deployment scheme and various performance indexes when expect total distance largest.

Scheme Number	SR	Number of Critical Spoofed Receivers	Number of Higher Power Forwarding Spoofed Receivers	$\sum d_{nm}^{Tr}\;(m)$	CSR	SSR
1	3	5	1	40,345.1	83.3%	100%
2	3	5	1	40,276.4	83.3%	100%
3	3	5	1	35,800.2	83.3%	100%
4	3	5	1	25,147.7	83.3%	100%
5	3	5	1	24,342.8	83.3%	100%
6	3	5	1	22,991.1	83.3%	100%
7	2	4	2	22,212.4	66.7%	100%
8	2	5	1	21,928.1	83.3%	100%

**Table 5:** Deployment scheme and various performance indexes when expect max of CSR and SSR.

Scheme Number	SR	Number of Critical Spoofed Receivers	Number of Higher Power Forwarding Spoofed Receivers	$\sum d_{nm}^{Tr}\;(m)$	CSR	SSR
1	2	6	0	13,803.5	100%	100%
2	2	6	0	5199.7	100%	100%

**Table 6:** Two-dimensional coordinates of victim receivers in MDPSA.

Victim Receiver	X (m)	Y (m)
$P_{r1}$	11	11
$P_{r2}$	511	−639
$P_{r3}$	911	11
$P_{r4}$	511	711
$P_{r5}$	−239	811
$P_{r6}$	−229	1011

**Table 7:** Plane coordinates and action radius of each spoofer.

Victim Receiver	X (m)	Y (m)	Action Radius
$P_{T1}$	−1106.3	−626.5	1286.3
$P_{T2}$	993.7	−1500	987.1
$P_{T3}$	3000	1293.5	2451.3
$P_{T4}$	225.7	910.8	348.3
$P_{T5}$	−397.8	684.6	202.9
$P_{T6}$	−1500	3000	2360.4

**Table 8:** Deployment scheme and various performance indexes in MDPSA.

Scheme Number	SR	Number of Critical Spoofed Receivers	Number of Higher Power Forwarding Spoofed Receivers	$\sum d_{nm}^{Tr}\;(m)$	CSR	SSR
1	6	6	0	70,106.4	100%	100%

## Data Availability

The data used in this research are not publicly available.
